# One-Carbon (Folate) Metabolism Pathway at Birth and Risk of Childhood Acute Lymphoblastic Leukemia: A Biomarker Study in Newborns

**DOI:** 10.3390/cancers15041011

**Published:** 2023-02-05

**Authors:** Catherine Metayer, Partow Imani, Sandrine Dudoit, Libby Morimoto, Xiaomei Ma, Joseph L. Wiemels, Lauren M. Petrick

**Affiliations:** 1 Division of Epidemiology, School of Public Health, University of California, Berkeley, CA 94704, USA; 2 Division of Biostatistics, School of Public Health, University of California, Berkeley, CA 94704, USA; 3 Department of Statistics, University of California, Berkeley, CA 94720, USA; 4 Department of Chronic Disease Epidemiology, Yale School of Public Health, New Haven, CT 06510, USA; 5 Center for Genetic Epidemiology, Keck School of Medicine, University of Southern California, Los Angeles, CA 90033, USA; 6 Department of Environmental Medicine and Public Health, Icahn School of Medicine, Mount Sinai, New York, NY 10029, USA; 7 The Bert Strassburger Metabolic Center, Sheba Medical Center, Tel-Hashomer, Ramat Gan 5211401, Israel

**Keywords:** childhood leukemia, folate, B-vitamins, newborn, metabolomics

## Abstract

**Simple Summary:**

Leukemia is the most frequent cancer in children. While cure rates have improved, many children will not survive, and of those who do, the majority experience lifelong complications. As a result, understanding what increases or decreases the risk of leukemia is important to inform prevention. Following on earlier observations that taking B-vitamins (such as folate) before and during pregnancy reduces the risk of childhood leukemia, we conducted a study to directly measure 11 nutrients in the folate metabolism pathway that is central to DNA integrity. These measurements were done in blood samples collected at birth among 122 children with leukemia and 122 healthy children, using novel laboratory techniques. Our data showed that none of these nutrients measured at birth (therefore representing levels within the last weeks of pregnancy) distinguished children who later contracted childhood leukemia. Whether levels of these nutrients may be important at the time of conception or during the first trimester, which are critical periods for fetal development, should be further investigated.

**Abstract:**

Leukemia is the most common cancer in children in industrialized countries, and its initiation often occurs prenatally. Folic acid is a key vitamin in the production and modification of DNA, and prenatal folic acid intake is known to reduce the risk of childhood leukemia. We characterized the one-carbon (folate) metabolism nutrients that may influence risk of childhood acute lymphoblastic leukemia (ALL) among 122 cases diagnosed at age 0–14 years during 1988–2011 and 122 controls matched on sex, age, and race/ethnicity. Using hydrophilic interaction chromatography (HILIC) applied to neonatal dried blood spots, we evaluated 11 folate pathway metabolites, overall and by sex, race/ethnicity, and age at diagnosis. To conduct the prediction analyses, the 244 samples were separated into learning (75%) and test (25%) sets, maintaining the matched pairings. The learning set was used to train classification methods which were evaluated on the test set. High classification error rates indicate that the folate pathway metabolites measured have little predictive capacity for pediatric ALL. In conclusion, the one-carbon metabolism nutrients measured at birth were unable to predict subsequent leukemia in children. These negative findings are reflective of the last weeks of pregnancy and our study does not address the impact of these nutrients at the time of conception or during the first trimester of pregnancy that are critical for the embryo’s DNA methylation programming.

## 1. Introduction

Leukemia is the most common cancer among children in industrialized countries, and its incidence has increased in the past 40 years [1,2]. Acute lymphoblastic leukemia (ALL) accounts for 80–85% of all leukemia diagnosed in children (0–14 years), and despite improvements in treatment, not all children have benefitted equally from a favorable prognosis and most survivors experience complications throughout their life [3]. Therefore, identification of modifiable risk factors of childhood leukemia is of high public heath relevance. Childhood ALL exhibits a peak in incidence at the age of 2–5 years, and several experiments have demonstrated that chromosomal abnormalities detected at diagnostic were also identified at birth (such as *ETV6-RUNX1*, *RUNX1-RUNX1T1*, and *PML-RARA* gene fusions, as well as high hyperdiploidy), providing strong support to the prenatal origin of many childhood leukemia subtypes [4,5,6,7,8]. In addition, a recent study on discordant twins for ALL supports a role for DNA methylation alterations in utero impacting leukemogenesis [9]; such DNA methylation patterns may be influenced by periconceptional nutrients including folate [10]. In epidemiologic studies using interview data, prenatal folic acid and vitamin intake from dietary sources and supplementation [11,12,13,14,15] has consistently emerged as a protective factor for childhood leukemia. B-vitamins (e.g., folate) and amino-acids (e.g., methionine) are important micronutrients in the one-carbon (folate) metabolism pathway that supports the maintenance of DNA and provision of methyl groups for epigenetic control of DNA expression. In contrary, lack of these nutrients can lead to cell damages including hematotoxicity, as best illustrated by one of the many side effects of methotrexate, a folate antagonist drug that decreases nucleic acid synthesis and thus compromises DNA synthesis, repair, and cellular replication [16]. However, despite a healthy folate intake during pregnancy overall, it is not clear whether inter-individual differences in the ability to metabolize folate may modulate risk. One-carbon metabolism nutrients support the embryo’s DNA programming mostly during preconception and the first weeks after conception. Folate levels during the remainder of the first trimester (referred to as early pregnancy) are critical for prevention of birth defects (neural, heart, and lip), yet it is unknown at which point these nutrients are critical for reduction in childhood leukemia risk, particularly towards the third trimester (referred to as late pregnancy). Direct measurement of downstream metabolites at birth can provide insight to this latter question. Our group has previously measured folate species in neonatal blood spots of 357 children with ALL and 405 controls and reported no association [17]. Little is known, however, about other key metabolites in the folate metabolism pathway. For example, homocysteine concentration is a marker of folate status (with an inverse relationship) and a metabolite closer to DNA methylation in the one-carbon cycle, and increased levels have been associated with cancers in adults [18,19]. Anecdotally, a child diagnosed with methylmalonic acidemia, a condition that leads to high levels of homocysteine, was reported to have multisystem disorders, including leukemia [20]. However, no population-based study has examined its potential role on development of childhood leukemia to date.

The goal of this study was to expand this characterization by using novel metabolomics techniques applied to neonatal blood specimens in a registry-based case/control study of childhood ALL conducted in California.

## 2. Materials and Methods

### 2.1. Study Population

We utilized archived neonatal dried blood specimens (DBS) from a subset of children diagnosed with ALL at age 0–14 years (International Classification of Disease for Oncology (ICD-O) codes 9820, 9823, 9826, 9827, 9831–9837, 9940, 9948) and controls identified through a California registry-based mother-child study linking electronic records from the cancer registry (1988–2011) with birth data from vital statistics. Of the 137 ALL cases originally identified, we included 122 children diagnosed at age 1 to 14 years and with available specimens from the California Department of Public Health biobank program. Controls were 1-to-1 matched on sex, year and month age of birth, and race/ethnicity.

### 2.2. Metabolite Extraction and Liquid Chromatography-High Resolution Mass Spectrometry (LC-HRMS) Analysis

DBS were included for the present analysis using a methodology developed by our team [21,22]. In short, DBS punches were extracted with 100 µL of water at room temperature (15 min, 1400 rpm), and a 5 μL aliquot was reserved for hemoglobin measurements to adjust for original blood volume [23]. 400 μL of acetonitrile containing isotopically labeled internal standards (IS) was added to the remaining aqueous solution containing the DBS punch, samples were agitated (1400 rpm, 37 °C, 1 h), and protein was precipitated at −20 °C for 30 min. The supernatant was evaporated to dryness and stored at −80 °C until analysis. Immediately prior to analysis, samples were reconstituted and analyzed through LC-HRMS with HILIC chromatography to capture folate pathway metabolites [24]. A pooled quality control (QC) sample prepared by combining aliquots of all of the sample extracts that were injected routinely throughout the run was used to monitor instrument stability and facilitate batch and run order correction [25]. The biological and QC samples were run in two sequential analyzed batches. The QC sample was run 30 times (15 in each batch) and at least once every 10 biological samples. Eleven folate pathway metabolites as well as 151 additional metabolites across a broad range of biological processes were identified considering retention time, accurate mass, and MS/MS matching, when available, with our in-house library of standards analyzed under the same LC-HRMS conditions using Profinder and Find by Formula software (Agilent Technologies, Santa Clara, CA, USA). The folate pathway metabolites measured in this study included methionine, betaine/valine, homocysteine, choline, taurine, dimethylglycine/2-aminoisobutyrate, cysteine, glycine, serine, adenosylhomocysteine, and cystathionine. Metabolites with multiple annotations are those that co-eluted and could not be distinguished.

### 2.3. Data Preprocessing

To facilitate downstream analyses that involve prediction assessment, the 244 samples (122 cases and 122 controls) were randomly separated into learning (75%; n = 186) and test (25%; n = 60) sets, maintaining the matched pairings. The QC samples were also randomly partitioned by batch (10 per batch for training and 5 per batch for testing). Random partitioning of the data in this manner maintains the study design and is standard when attempting to estimate predictive ability. It allows for training of classifiers on a learning data set and then evaluation of the candidate classifiers on an independent test data set. This extra precaution is to ensure that prediction estimates are not overly optimistic due to over-fitting that can occur when evaluating classifiers on the same data that they were trained on. Until otherwise mentioned, this next portion describes analyses performed on the learning set. The 162 metabolites were then filtered based on Interclass Correlation Coefficient (ICC) and percent missing. To perform the ICC filtering, a random effects model was fit separately for each metabolite using the QC samples as technical replicates in order to estimate the proportion of the variance that was due to technical effects as opposed to biological effects [26]. This was performed separately for each batch and, using an empirical ICC cutoff of 0.2, resulted in the removal of three metabolites for a remainder of 159. Following this, a two-step process was used to remove metabolites with too many missing values for reasonable imputation. The percent of missing values for each metabolite across all study samples was calculated, and any metabolite with more than 60% missing values was removed. For any metabolite with 30–60% missing values, a Fisher’s exact test was used to check for differential missingness by case/control status [26]. This two-step process allows for retaining metabolites with lower percentages of missing values where missing values appear to be related to the biological condition of interest. As there did not appear to be evidence for differential missingness, all metabolites with more than 30% missing values were removed, leaving all 11 folate pathway metabolites and an additional 137 metabolites in the data set for analysis. Imputation was then performed using the k-nearest neighbor algorithm with the number of neighbors set to 5 [27]. Finally, the scone package was used to evaluate the relative ability of various normalization schemes to remove unwanted variation while maintaining the biological signal of interest [28]. In addition to the imputed data, scone was provided with batch, case/control status, and a QC matrix, which included variables that were thought to help control for various sources of unwanted variation, including blood spot age, run order, hemoglobin, and the newborn’s age (in hours) when the sample was taken. Scone was also provided with five scaling options [identity (no scaling), DESeq, Upper Quartile (UQ), Variance Stabilization (VSN), and Trimmed Mean of M-values (TMM)] [29,30,31,32]. Based on the scone performance measures, UQ scaling while adjusting for case/control status, batch, and the full QC matrix was selected and implemented to acquire the normalized data.

### 2.4. Statistical Analysis

Using the normalized data, the abundances of the 11 folate pathway features were visualized univariately as well as by using dimensionality reduction techniques such as Principal Components Analysis (PCA) and Uniform Manifold Approximation and Projection (UMAP). A prediction framework using a range of methods was then used to try to evaluate the predictive ability of the folate pathway metabolites on childhood ALL. For each method, the normalized abundances of the 11 folate pathway metabolites as well as sociodemographic and reproductive factors that have been associated with ALL risk [33] were provided as input. These covariates are sex (binary), race/ethnicity (categorical), mode of delivery (binary: vaginal vs. cesarean), mom’s education level (categorical), and birth weight conditional on gestational age (binary: above or below the 90th percentile using INTERGROWTH 21 standards) [34]. The candidate class prediction methods used were Random Forests (RF), Linear Discriminant Analysis (LDA), and k-Nearest Neighbors (kNN) using a range of number of neighbors. In this case, kNN refers to the classification algorithm rather than the imputation algorithm. In order to select the optimal number of neighbors k in the kNN algorithm, Monte Carlo cross-validation was used for a range of k values from 3 to 25. For each iteration, the data were split into 60% training and 40% validation sets, each classifier was trained on the training set, and classification errors were calculated on the validation set. The kNN classifier with the number of neighbors k which resulted in the lowest classification error rate was selected for future evaluation in the testing set along with the LDA and RF predictors. These three classifiers were then trained on the full learning set. These analyses were repeated on stratified datasets by sex, race/ethnicity, and age at diagnosis (≤2 years vs. >2 years), in case heterogeneities between the groups were masking the signal. The sex and race/ethnicity variables were dropped in their respective stratified analyses.

### 2.5. Testing Data Analysis

Once the candidate classifiers were selected using the learning set, the independent test set was used to acquire an unbiased estimate of the various predictors’ classification error rates. The preprocessing pipeline of the testing data was identical to that of the learning data mentioned previously with the exception that any metabolites of interest were retained even if they failed one of the filtering steps. This was the case for 2 metabolites, one of which was a folate pathway metabolite, leaving 151 metabolites in the testing data set for analysis. Following kNN imputation with the number of neighbors set to 5, scone evaluation selected DESeq scaling adjusting for case/control status, batch, and the full QC matrix. Using the normalized data set, each of the classifiers trained on and selected from the learning set was applied to predict case/control status on the testing set and classification error rates were reported.

## 3. Results

**Figure 1:**
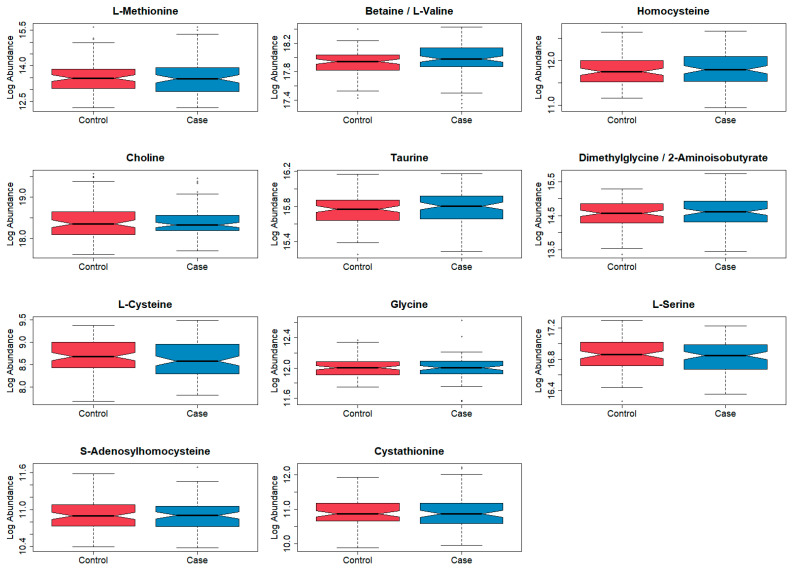
Boxplots of log abundances for each folate pathway metabolite, stratified by case/control status, following filtering and normalization. Blue = cases (n = 92) and red = controls (n = 92). Note the different y-axis scales.

**Figure 2:**
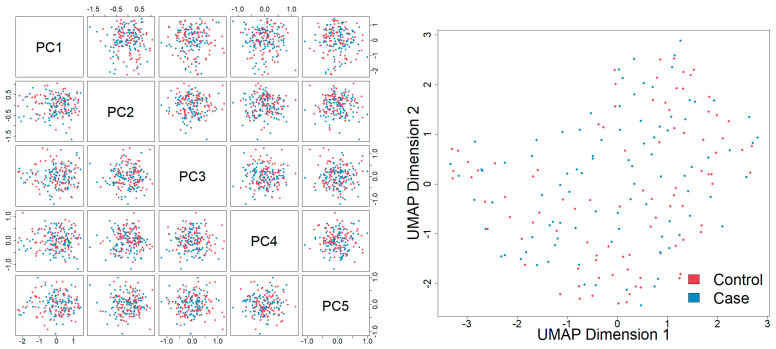
(**Left**) Pairwise plots of the first 5 principal components from PCA on only the 11 folate metabolites and all subjects, with plotting symbols for subjects colored by case/control status. (**Right**) Results of UMAP on only the 11 folate metabolites and all subjects plotted and colored by case/control status. Blue = cases (n = 92) and pink = controls (n = 92).

**Figure 3:**
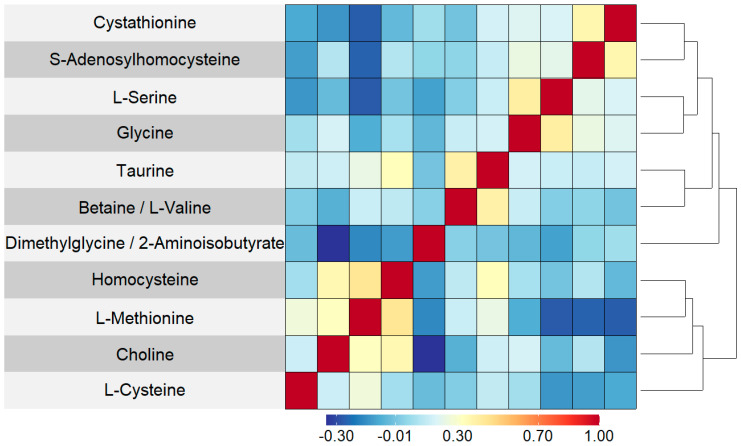
Pseudo-color image of Spearman correlation matrix for 11 folate pathway features, with rows and columns ordered by complete linkage hierarchical clustering. A darker red indicated a stronger positive correlation and a darker blue indicated a stronger negative correlation. As it is a symmetric matrix, the columns correspond to the same set and ordering of metabolites as the rows.

Table 1 shows demographic summary statistics for cases and controls separately. Visualizations of these variables stratified by case and control are also shown in Supplementary Figures S1–S7. There was evidence of a difference in the distributions of the timing of the newborn’s blood spot collection after their birth (in hours) between cases and controls, which was a variable adjusted for during the normalization process. There was no correlation between blood spot age (defined as the number of years between the sample collection and processing at the laboratory) and folate pathway metabolite levels (spearman correlation coefficients ranged from −0.04 to 0.06) (Table S1). Boxplots of the log abundances of each folate pathway metabolite showed no noticeable differences in median values between cases and controls for any of the folate pathway metabolites (Figure 1). Visualizations of the folate metabolite data using dimensionality reduction on all subjects are presented in Figure 2. Specifically, Figure 2 shows pairwise plots of the first five principal components from PCA as well as the two components from UMAP, with each point colored by case/control status. Figure 2 did not reveal any clustering by case/control status. In addition, the folate pathway metabolites themselves did not appear to be correlated, as indicated by the pseudo-color image of the Spearman correlation matrix with rows/columns ordered by hierarchical clustering (Figure 3). When comparing the folate pathway features to the other metabolites present in the data set, the folate pathway metabolites did not appear to cluster together (Figure S8). Lastly, Table 2 lists the classification error rates of the prediction algorithms on the hold-out testing set, which shows no evidence of predictive ability of the folate pathway metabolites for childhood ALL (a classification error rate of 0.5 corresponding to chance alone).

## 4. Discussion

Epidemiologic studies have reported a reduced risk of childhood leukemia following self-reported prenatal folate and vitamin intake from supplementation and diet [11,12,13,14,15], yet the mechanistic pathways and the windows of sensitivity during the prenatal period are not well understood. Our study characterized downstream one-carbon (folate) related metabolites at birth and found no predictive value of 11 nutrients in the development of childhood ALL. Null findings were observed overall, as well as in stratified analyses by sex, race/ethnicity, and age at diagnosis. Our interest in examining these groups stemmed from the known higher incidence of childhood leukemia in boys and Latinx children [1,2], and the difference in molecular characteristics by age at onset [35,36]. Our observations are consistent with a previous report from our group using an independent study population of childhood ALL cases and controls showing no association between neonatal hemoglobin-normalized folate concentrations measured by the *Lactobacillus casei* microbiologic growth assay and risk of childhood ALL (n = 313) [17]. To our knowledge, no other childhood leukemia studies have directly measured downstream folate pathway metabolites, neither at birth nor in cord blood or pregnancy blood samples. In a cohort study conducted in England [37], DNA methylation levels measured in cord blood samples were associated with self-reported folic acid supplementation during pregnancy. However, these folate-associated CpG sites did not overlap with known ALL-associated CpG sites, providing no support that folic acid acts via DNA methylation in preventing childhood leukemia [37]. Most other biomarker studies to date have examined the role of genetic variants in the folate pathway. Meta-analyses have consistently reported that the MTHFR C677T polymorphism, which regulates folate and homocysteine metabolisms, is associated with childhood ALL risk. However, homocysteine as measured in our study was not predictive of childhood ALL; the evidence for other folate-related genes is less strong, mostly due to lack of studies with sufficient sample sizes and validation in independent test sets [38,39,40]. Measuring the downstream metabolites in the folate pathway should account for inter-individual variations that may exist in terms of genetic susceptibility, methylation processes, and other lifestyle factors.

Our study focused on the neonatal period, as a surrogate of last weeks of the pregnancy, and negative findings may be explained by the fact that nearly all women in the US take prenatal vitamins by the end of their pregnancy [41], limiting our ability to find case/control differences in newborn levels of one-carbon nutrients; studies implemented in other countries where prenatal folate/vitamin supplementation is less complete than in the US could be informative. Also, there is a chance that the null findings could be attributed to our relatively small sample size. Alternatively, the timing of a potential impact of folate-related nutrients on blood cell development and epigenome at large in the fetus may occur in early pregnancy, as shown in studies of nutritionally deprived women during early pregnancy [42,43], although no studies to date have directly tested this yet for childhood leukemia. Our group has recently published results on the relationship of DNA methylation patterns associated with periconception folate intake in children with ALL vs. healthy controls [10]. This epigenome-wide association analysis revealed a differentially methylated region (DMR) in the promoter region of *DUSP22*, a protein phosphatase and known tumor suppressor, in response to total and food periconceptional folate intake. While the direction of effect was the same between cases and controls, the strength of association was greater for cases. Additional relationships between dietary periconceptional folate and DNA methylation by case status were identified via differentially methylated probes in genes *CERK, CUTA, WDFY4, SART1, TCF20,* and *ERGIC1*. These results lend support for a role of folate as a drive of early pregnancy changes in DNA methylation that affect future ALL development.

Our case/control study has several strengths. Childhood leukemia is a rare disease, and investigating its etiology is hampered with several methodological challenges. Here, we assembled 122 cases and 122 controls with unique access to blood specimens collected at birth to better characterize the perinatal window of susceptibility. The use of these archived neonatal blood specimens allowed the examination of events before the leukemia occurred, thus preserving temporality in investigating causation. The cases and controls in this study were age-matched by year and month of birth to reduce any potential bias in the results due to storage conditions. Nevertheless, all metabolites were adjusted for ‘blood spot age’ as well as ‘hemoglobin’ which also accounts for storage aging factors [44]. Lastly, we confirmed that storage conditions likely did not bias our results since blood spot age was not correlated with the levels of metabolites in cases and controls. We developed and validated laboratory methods to measure exposomic features with extremely small amounts of blood from the neonatal spots. Our statistical approach focused on the predictive ability of the set of folate-related metabolites rather than hypothesis testing for individual metabolites and assessed the performance of three distinct classes of predictors using sample-splitting. Our data are derived from a registry-based study with no contact with participants, therefore reducing the potential for differential participation between cases and controls. The subset included in this study represented socio-demographic and reproductive characteristics similar to the larger source California registry-based study [45]. Folate deficiency can cause chromosome breakage [46], and DNA-damaging agents have been associated with childhood-leukemia-harboring chromosome structural abnormalities (such as *KMT2A* gene fusion in infant leukemia, and *ETV6-RUNX1* translocation) [36,47,48]. However, information on molecular subtypes of leukemia was not available in this series derived from a cancer registry database. Additionally, the type of specimen available at birth (i.e., archived dried blood spot) does not allow for measuring metabolites separately in different cellular blood components (like lymphocytes, monocytes, etc.). The statistical methods used did not utilize any information about the cyclic structure of the folate pathway; future development of statistical methods to address this issue could improve predictive performance. The analyses were limited to only 11 folate pathway metabolites with semi-quantitative measures.

## 5. Conclusions

In our study, the 11 analytes involved in the one-carbon (folate) metabolism pathway that were measured in newborn blood specimens did not appear to predict subsequent ALL in children. These negative findings are reflective of the last weeks of pregnancy, and alteration of the DNA methylation under the influence of various nutrients, including folic acid and their metabolites, during early gestation could be a more important factor.

## Figures and Tables

**Table 1:** Demographic summary statistics by case/control status.

	Cases	Controls	*p*-Value *
Sex	n (%)	n (%)	1
Female	53 (43.4)	53 (43.4)	
Male	69 (56.6)	69 (56.6)	
Child’s age at diagnosis (years)			
Min	1.00	n/a	n/a
Mean	3.07		
Median	3.00		
Max	7.00		
Race/ethnicity	n (%)	n (%)	0.42
Non-Latinx white	35 (28.7)	38 (31.1)	
Latinx	67 (54.9)	71 (58.2)	
Other	20 (16.4)	13 (10.7)	
Mode of delivery	n (%)	n (%)	0.10
Vaginal	75 (61.5)	88 (72.1)	
Cesarean	47 (38.5)	34 (27.9)	
Birthweight conditioned on gestational age (percentile)			
Min	1.31	0.08	0.74
Mean	60.38	59.65	
Median	66.01	65.23	
Max	99.97	99.81	
Time of blood draw relative to newborn’s birth (hours)			
Min	<1	12	0.05
Mean	35	31	
Median	29	27	
Max	131	120	
Blood spot age at the time of the laboratory analyses (years)			
Min	11	11	1
Mean	15.33	15.33	
Median	15	15	
Max	19	19	

* The *p*-value corresponds to a test for differences between case and control groups. For binary variables, Fisher’s exact test was used to test for differences between groups; for categorical variables, a Chi-squared test was used; and for continuous variables, a Wilcoxon rank sum test was used.

**Table 2:** Classification error rates for each candidate class predictor after being trained on the learning set and then evaluated on the hold-out test set.

	Total	Females	Males	≤2 Years Old at Diagnosis	>2 Years Old at Diagnosis	White	Latinx
	n = 54	n = 17	n = 37	n = 25	n = 29	n = 17	n = 28
kNN	0.5185	0.5882	0.4595	0.56	0.4482	0.4706	0.3929
LDA	0.5556	0.5882	0.4595	0.4	0.5172	0.5882	0.3571
RF	0.537	0.5882	0.4595	0.56	0.5517	0.5294	0.3929

Abbreviations: kNN = k-Nearest Neighbors; LDA = Linear Discriminate Analysis; RF = Random Forest.

## Data Availability

The data presented in this study are available upon request from the corresponding author and after approval from the California Health and Human Services. The data are not publicly available due to California Health and Human Services restrictions.

## Supplementary Materials

The following supporting information can be downloaded at: https://www.mdpi.com/article/10.3390/cancers15041011/s1, Figure S1: Mosaic plot of 2 × 2 contingency table for joint distribution of case/control status and sex; Figure S2: Barplot for distribution of age at diagnosis (in years) for cases; Figure S3: Mosaic plot of 2 × 3 contingency table for joint distribution of case/control status and race; Figure S4: Mosaic plot of 2 × 2 contingency table for joint distribution of case/control status and mode of delivery; Figure S5: Boxplots of weight percentiles conditional on gestational age, stratified by case/control status. Calculated using the INTERGROWTH-21st standards; Figure S6: Boxplots of age at blood collection stratified by case/control status; Figure S7: Boxplots of number of years from sample collection to sample processing, stratified by case/control status; Figure S8: Pseudo-color image of Spearman correlation matrix for all 148 metabolites, with rows and columns ordered by complete linkage hierarchical clustering. Darker red indicated a stronger positive correlation and a darker blue indicates a stronger negative correlation; Table S1: Spearman correlation coefficients between blood specimen age (defined as the numbers of years between the sample collection and processing at the laboratory) and measured metabolites in the folate pathway.
