# Supplementary material for: One-Carbon (Folate) Metabolism Pathway at Birth and Risk of Childhood Acute Lymphoblastic Leukemia: A Biomarker Study in Newborns
Source: Cancers (Basel). 2023 Feb 5;15(4):1011. doi: 10.3390/cancers15041011 (PMC9953980; doi:10.3390/cancers15041011)
Supplement: Supplementary file 1 [file cancers-15-01011-s001.zip › cancers-2176351-supplementary.pdf]

One-carbon (folate) metabolism pathway and risk of childhood acute lymphoblastic leukemia at birth: a biomarker study in newborns

Supplementary Materials:

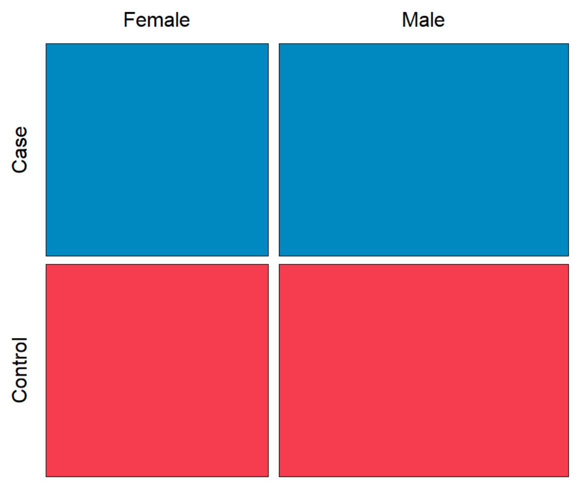

Figure S1. Mosaic plot of 2x2 contingency table for joint distribution of case/control status and sex.

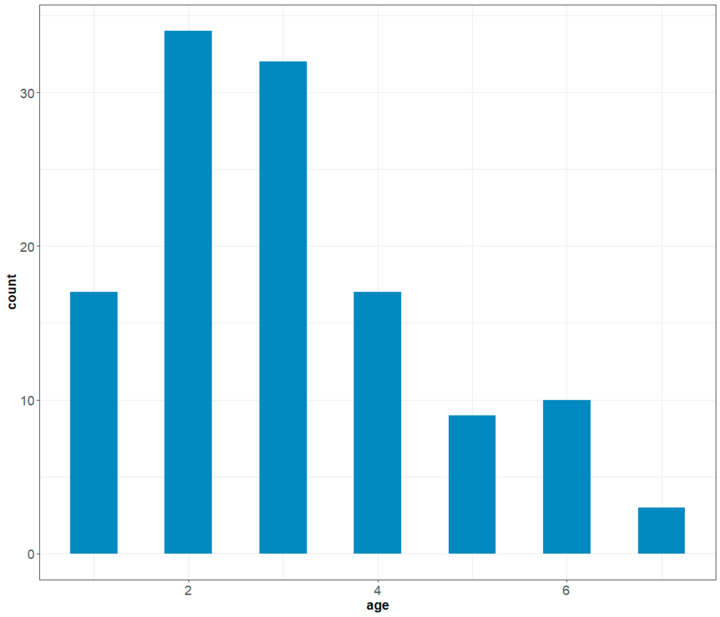

Figure S2. Barplot for distribution of age at diagnosis (in years) for cases.

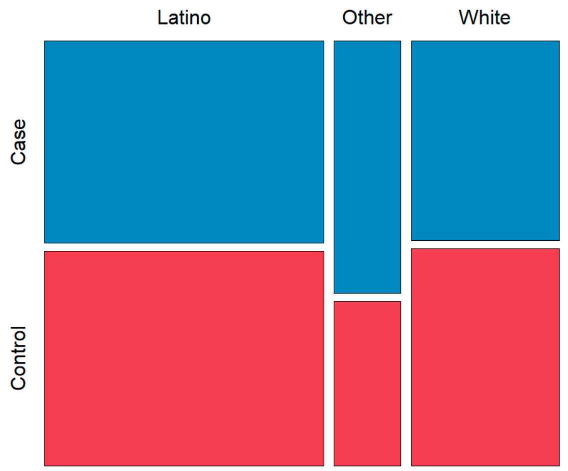

Figure S3. Mosaic plot of 2x3 contingency table for joint distribution of case/control status and race.

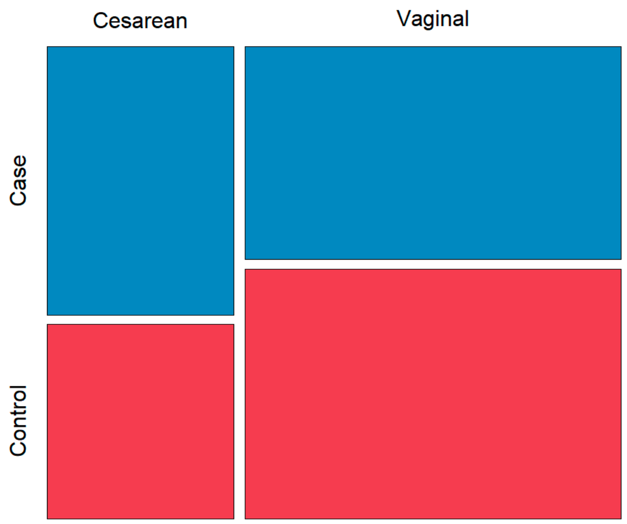

Figure S4. Mosaic plot of 2x2 contingency table for joint distribution of case/control status and mode of delivery.

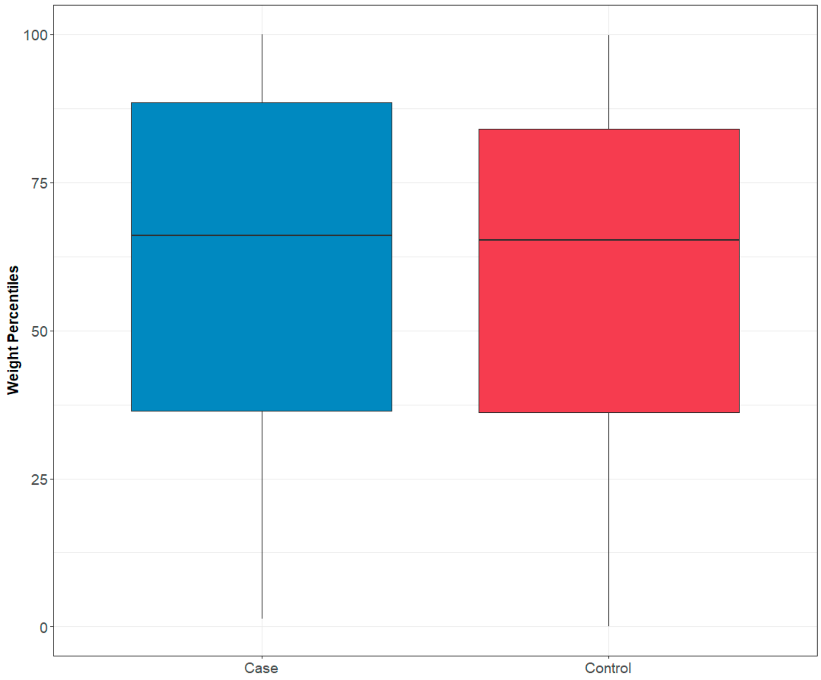

Figure S5. Boxplots of weight percentiles conditional on gestational age, stratified by case/control status. Calculated using the INTERGROWTH-21st standards.

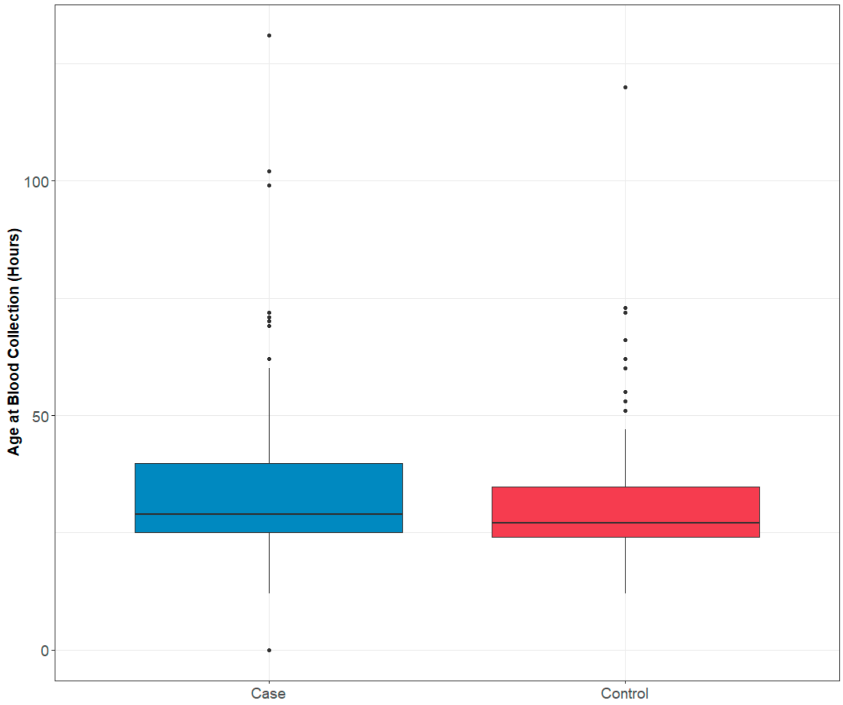

Figure S6. Boxplots of age at blood collection stratified by case/control status.

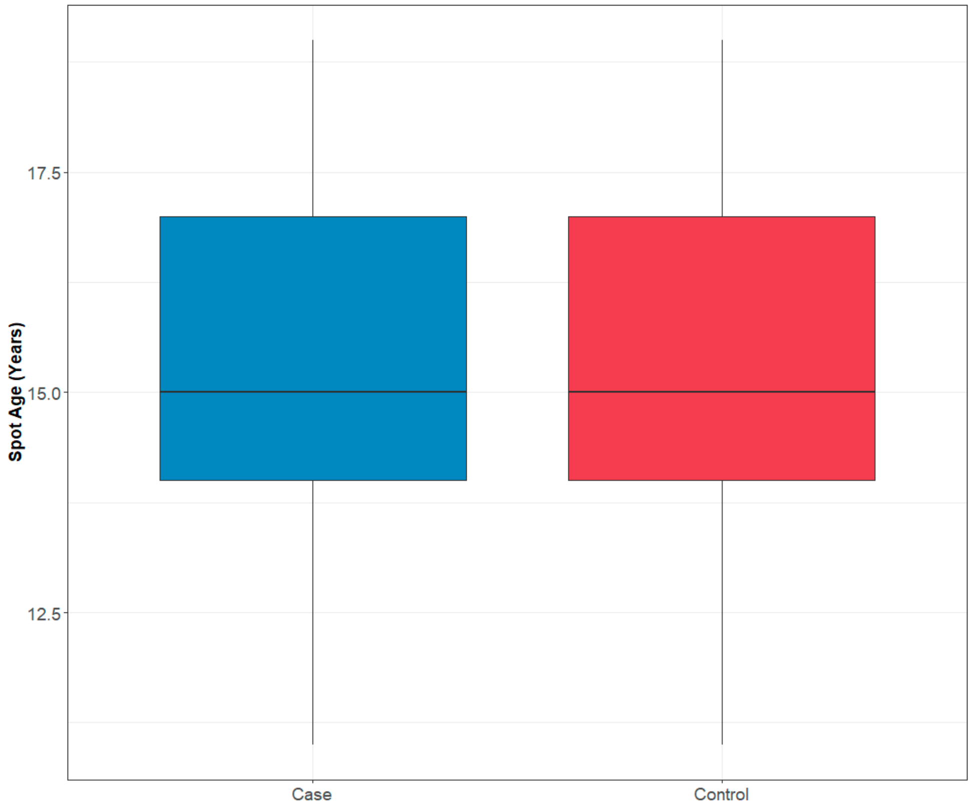

Figure S7. Boxplots of number of years from sample collection to sample processing, stratified by case/control status.

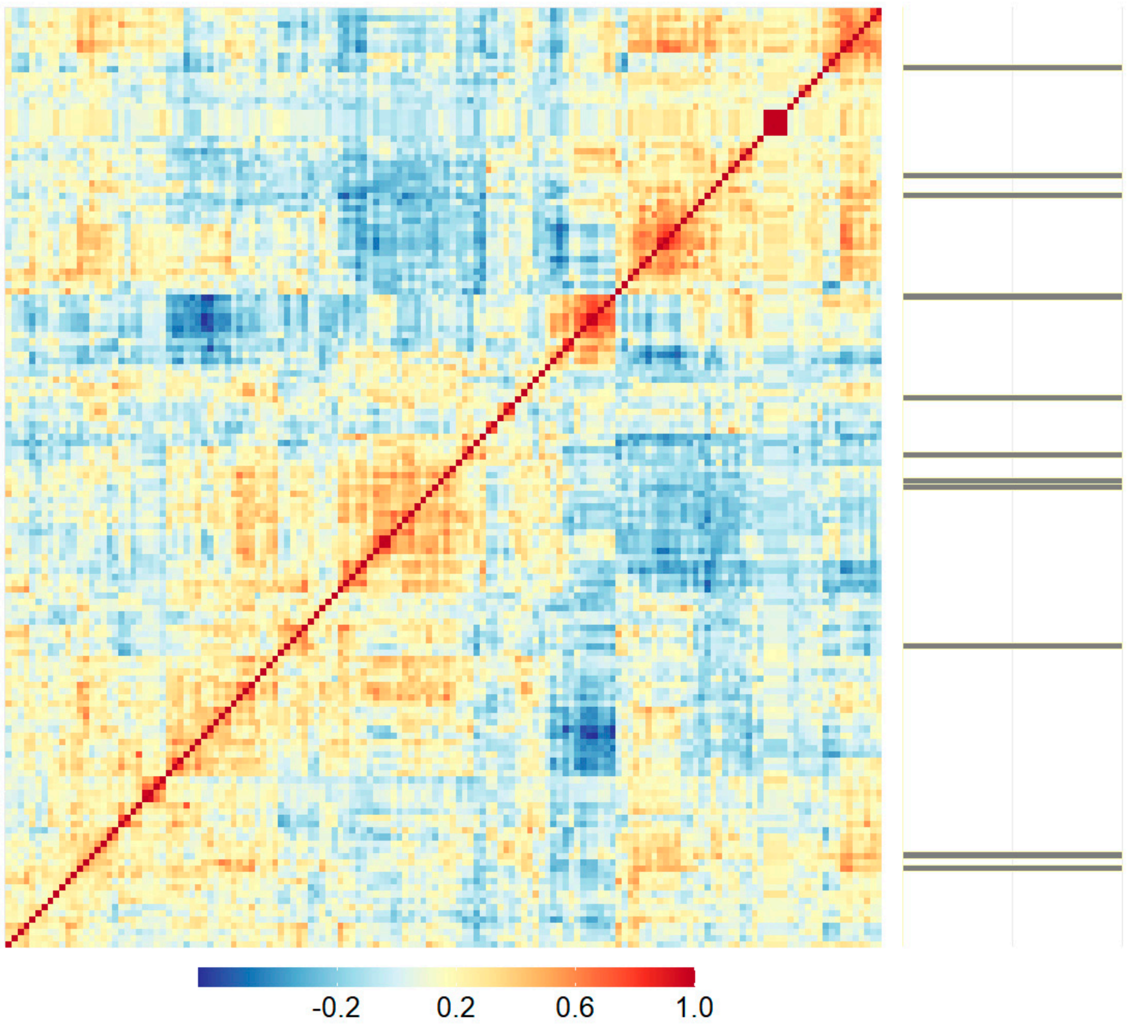

Figure S8. Pseudo-color image of Spearman correlation matrix for all 148 metabolites, with rows and columns ordered by complete linkage hierarchical clustering. Darker red indicated a stronger positive correlation and a darker blue indicates a stronger negative correlation.

Table S1. Spearman correlation coefficients between blood specimen age (defined as the numbers of years between the sample collection and processing at the laboratory) and measured metabolites in the folate pathway.

Metabolites	Spearman Correlation Coefficient
L-Methionine	0.057
Betaine / L-Valine	-0.002
Homocysteine	-0.016
Choline	0.043
Taurine	0.013
Dimethylglycine / 2-Aminoisobutyrate	0.003
L-Cysteine	0.027
Glycine	-0.021
L-Serine	0.009
S-Adenosylhomocysteine	-0.022
Cystathionine	-0.031
